# The Beneficial Impact of Mineral Content in Spent-Coffee-Ground-Derived Hard Carbon on Sodium-Ion Storage

**DOI:** 10.3390/ma17051016

**Published:** 2024-02-22

**Authors:** Sonya Harizanova, Ivan Uzunov, Lyubomir Aleksandrov, Maria Shipochka, Ivanka Spassova, Mariya Kalapsazova

**Affiliations:** Institute of General and Inorganic Chemistry, Bulgarian Academy of Sciences, 1113 Sofia, Bulgaria; sonya@svr.igic.bas.bg (S.H.); uzunov_iv@svr.igic.bas.bg (I.U.); lubomir@svr.igic.bas.bg (L.A.); shipochka@svr.igic.bas.bg (M.S.); ispasova@svr.igic.bas.bg (I.S.)

**Keywords:** hard carbon, spent coffee grounds, sodium-ion storage, mineral content, albite

## Abstract

The key technological implementation of sodium-ion batteries is converting biomass-derived hard carbons into effective anode materials. This becomes feasible if appropriate knowledge of the relations between the structure of carbonized biomass products, the mineral ash content in them, and Na storage properties is gained. In this study, we examine the simultaneous impact of the ash phase composition and carbon structure on the Na storage properties of hard carbons derived from spent coffee grounds (SCGs). The carbon structure is modified using the pre-carbonization of SCGs at 750 °C, followed by annealing at 1100 °C in an Ar atmosphere. Two variants of the pre-carbonization procedure are adopted: the pre-carbonization of SCGs in a fixed bed and CO_2_ flow. For the sake of comparison, the pre-carbonized products are chemically treated to remove the ash content. The Na storage performance of SCG-derived carbons is examined in model two and three Na-ion cells. It was found that ash-containing carbons outperformed the ash-free analogs with respect to cycling stability, Coulombic efficiency, and rate capability. The enhanced performance is explained in terms of the modification of the carbon surface by ash phases (mainly albite) and its interaction with the electrolyte, which is monitored by ex situ XPS.

## 1. Introduction

The pursuit of climate neutrality is the main task of the large-scale implementation of renewable energy [1]. Since renewable energy is dependent on meteorological conditions, devices are needed to store and send the generated energy when needed [2]. Rechargeable lithium-ion batteries (LIBs) appear attractive due to their high energy density and good cycling stability. Nevertheless, LIBs could not meet the requirements for large-scale stationary energy storage systems due to the limited and uneven distribution of lithium resources, as well as the rapidly increasing price. For this reason, it is necessary to develop non-lithium energy storage technologies that can replace LIBs for large-scale applications. Although several forms of energy storage devices have been commercialized, the development of new ones that are more cost-effective and more sustainable than existing ones is imperative [1,2]. One of the most attractive alternatives among emerging technologies for large-scale energy storage is that of sodium-ion batteries (SIBs) [1,3,4]. Although SIBs are on the cusp of commercialization, the key to their technological implementation is finding an appropriate anode material.

In recent years, many anode materials have been tried for SIBs, among which non-graphitized carbons (amorphous carbons, soft carbons, and hard carbons) stand out due to their low cost, structural stability, and ease of industrial production. Currently, most amorphous carbons are industrially synthesized from coal petrochemical products [5], but the growing demand for the sustainability and recycling of materials draws attention to agricultural waste and so-called waste biomass like avocado peels [6], peanut shells [7], and corn straw pith [8]. Among the leading sources of bio-waste is coffee [9], with Indonesia as the largest coffee producer [10]. The statistics of the International Coffee Organization show that for 2020/2021, the world consumption of coffee was almost 10 tons, with the largest amount in Europe—about 33% [11]. Each ton of green coffee produces around 650 kg of spent coffee grounds (SCGs) [9,12], and these large amounts of waste require strategies to be found and developed for its reuse. Coffee waste is used for the production of enzymes, bioactive components, and biofuels [9], and in recent years, it has also attracted attention as a raw material for the production of electrode materials for SIBs [13,14]. SCGs are rich in many organic compounds and, during pyrolysis, yield hard carbons (HCs) whose structure and properties depend on the carbonization procedure. The potential of SCGs as an anode for SIBs was first demonstrated by Gao et al., where a high specific capacity and unsatisfactory rate capability were found [15]. To improve the electrochemical performance of SCGs, the combination of pyrolysis with chemical treatment has been applied [16,17]. The most frequently used chemical reagents are H_3_PO_4_ and/or HCl, which ensure the hydrolysis of the pretreated biomass [18]. As an activation agent, KOH is utilized with the aim of enlarging the pore size of coffee-derived carbons [19,20]. Hydrothermal carbonization (HTC) is another step used to enhance the electrochemical performance of carbons [21]. The final temperature of carbonization also affects the Na-storage properties [17]. The most widely applied carbonization process for obtaining hard carbon is one-step pyrolysis at temperatures between 900 and 1600 °C [7,8]. Although most studies indicate that carbonized temperatures between 800 and 970 °C are sufficient to form electrochemically active SCG-derived carbons [15,19,20], some studies propose higher temperatures (such as 2000 °C) as most suitable [22]. These discrepancies come from the fact that one-step and two-step pre-carbonization procedures are used. Some research groups have found that the two-step heat treatment enhances the electrochemical performance of biomass-derived hard carbon [23,24]. According to Zhang et al., mineral impurities (coming from ash content in pinecone biomass, such as Mg, P, K, S, Ca, and Si) may occupy some active sites, as a result of which the insertion of sodium ions becomes difficult [25]. They found out that the pre-pyrolyzed samples at 500 °C after treatment with KOH and HCl and additional carbonization at 1400 °C showed a better cycling performance in comparison with unwashed samples, i.e., 328 and 299 mAh g^−1^ after 100 cycles, respectively [25]. All the aforementioned chemical pre-treatments aim to modify the carbon content, but they are usually non-clean techniques and make biomass-derived carbons more expensive.

In addition to the organic components, the SCGs contain a relatively low number of inorganic components composed of potassium, magnesium, phosphorus, calcium, etc. (i.e., around 2 wt.%) [26,27,28]. During the pyrolysis of SCGs, the inorganic components are transformed, forming a variety of ash phases, namely potassium magnesium silicate, magnesium oxide, dicalcium silicate, pyrophyllite (Al_2_Si_4_O_10_(OH)_2_), etc. [29,30,31]. The impact of ash phases on the electrochemical properties of biomass-derived hard carbon is a subject of intensive study nowadays, but a unified picture is missed [32,33,34]. Most of the research groups highlight a strong requirement for the removal of mineral ash from biomass (before or after carbonization) in order to improve the Na storage properties of hard carbon [32,35]. However, some studies report an augmentation in the Na^+^ diffusion kinetics due to the inclusion of ash-forming elements in the graphitic layers [36]. The inconsistency in these reported data is a consequence of the complexity and variety of the ash phase composition, which, in its turn, depends on the nature of biomass sources. Understanding the role of the ash phase content is also important from the point of view of avoiding the use of expensive and pollutant chemical pre-treatment procedures during the production of carbonaceous electrodes from bio-waste. 

In this study, we examine the simultaneous impact of the ash phase composition and carbon structure on the Na storage properties of hard carbons derived from spent coffee grounds (SCGs). The carbon structure is modified using the pre-carbonization of SCGs at 750 °C, followed by annealing at 1100 °C in an Ar atmosphere. Two variants of the pre-carbonization procedure are adopted: the pre-carbonization of SCGs in a fixed bed and CO_2_ flow. For the sake of comparison, the pre-carbonized products are chemically treated to remove the ash content. The Na storage performance of SCG-derived carbons is examined in model two and three Na-ion cells. The electrode–electrolyte interaction after the electrochemical reaction is monitored by ex situ XPS.

## 2. Materials and Methods

### 2.1. Synthesis Procedure

**Figure 1:**
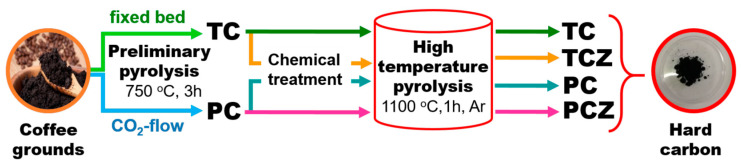
Schematic representation of the preparation of hard carbon from spent coffee.

The spent coffee grounds (SCGs) were obtained from leftovers of freshly ground coffee beans boiled in a coffee machine. The wet waste was dried at room temperature. The hard carbons were synthesized by the pyrolysis of dried waste following two temperature steps (Figure 1)—the coffee waste was first pre-carbonized at 750 °C and then annealed at 1100 °C in an Ar flow. The pre-carbonization of SCGs was performed in the following two ways: pyrolysis in a fixed bed (i.e., pyrolysis in an atmosphere of evolved gasses and lack of oxygen) and pyrolysis in a CO_2_ flow. Two types of pre-carbonized samples were denoted as TC and PC, respectively. After pyrolysis, the solid residues were divided into two parts, one of which was chemically treated to remove the ash content, while the other part remained untreated. The chemical treatment involves the successive boiling of TC and PC samples in HCl, HNO_3_, and NaOH for 3 h under reflux conditions. After each treatment, the samples were washed with deionized water until pH = 7 and dried. All chemically treated samples (TC and PC) were signified with an additional letter Z (i.e., TCZ and PCZ). The final temperature treatment was carried out at 1100 °C for 1 h, at a constant Ar flow of 0.3 ln min^−1^. The tube furnace equipped with a gas supply system (Nabertherm, Lilienthal, Germany) was used for this procedure. After treatment at 1100 °C, the products were slowly cooled down to an ambient temperature in an Ar atmosphere. These carbonaceous materials were ground in a planetary ball-milling system (Pulverisette 6, Fritsch, Idar-Oberstein, Germany) for 3 h, and a fraction with a 0.1 mm size was collected and used for characterization. As a result of these procedures, the pre-carbonized samples at 750 °C are denoted as TC-8, TCZ-8, PC-8, and PCZ-8, while the pyrolyzed samples at 1100 °C are TC-11, TCZ-11, PC-11, and PCZ-11, respectively.

### 2.2. Characterization Methods

The moisture and ash content of the air-dried SCGs were determined in accordance with TAPPI T-550 and T-211 standards, respectively [37].

Differential thermal analysis with thermogravimetry (DTA/TGA) of the SCGs and carbonaceous materials was performed with a Seteram Labsysis Evo 1600 instrument (SETARAM Company, Caluire, France) in the temperature range from room temperature up to 1000 °C at a heating rate of 5 K min^−1^ in oxygen or argon atmosphere.

The X-ray powder diffraction patterns of ash and carbonaceous materials were collected on the diffractometer Bruker D8 Advance (Bruker Corporation, Karlsruhe, Germany) with Cu Kα radiation.

The morphology of hard carbons was monitored by scanning electron microscope (SEM, JEOL JSM 6390, Tokyo, Japan) equipped with an energy-dispersive X-ray spectrometer (EDS, AZtec, Oxford Instruments, Abingdon, UK).

The elemental analysis (determination of C, H, N, S) was performed on a Vario Macro Cube (Elementar Analyzensysteme GmbH, Langenselbold, Germany). Oxygen content is determined by the difference.

Textural characterization of the carbon materials was carried out by low-temperature nitrogen adsorption at −196 °C using a Quantachrome NOVA 1200e (AntonPaar Quanta Tech Inc., Boynton Beach, FL, USA) instrument. Before the experiments, the samples were outgassed under a vacuum overnight. The adsorption–desorption isotherms are used for the evaluation of the specific surface area (by the BET method) and the pore volumes (according to the Gurvitch rule). The pore-size distributions were made by DFT (using the slit pore NLDFT equilibrium model).

The film composition and electronic structure were investigated by X-ray photoelectron spectroscopy (XPS). The measurements were carried out on an AXIS Supra electron- spectrometer (Kratos Analitycal Ltd., Manchester, UK) using monochromatic AlKα radiation with a photon energy of 1486.6 eV and a charge neutralization system. The binding energies (BEs) were determined with an accuracy of ±0.1 eV. The chemical composition in the depth of the films was determined by monitoring the areas and binding energies of C 1s, O 1s, N 1s, Ca 2p, Si 2p, Mg 2p, and K 2p photoelectron peaks. Using the commercial data-processing software of Kratos Analytical Ltd. (ESCApe™ 1.2.0.1325, Kratos Analytical A Shimadzu, Stretford, UK), the concentrations of the different chemical elements (in atomic %) were calculated by normalizing the areas of the photoelectron peaks to their relative sensitivity factors.

### 2.3. Electrode Preparation and Electrochemical Characterization

The electrodes were prepared by mixing 80% hard carbons with 10% carbon black (Super C65, TiMCAL Ltd., Bodio, Switzerland) and 10% carboxymethyl cellulose (CMC, Merck, St. Louis, MO, USA) in ultra-pure water. The mixture was homogenized in a planetary centrifugal mixer (Thinky Corporation, Tokyo, Japan) for 15 min at 2000 rpm. The obtained slurry was cast on conducted carbon-coated aluminum foil using a Doctor Blade film coater (Proceq SA, Scherzenbach, Switzerland), followed by vacuum drying at 80 °C overnight. The disk electrodes with a diameter of 10 mm were cut, pressed, and dried at 120 °C under a vacuum. The mass loading of active material varied between 2.8 mg and 3.2 mg.

All electrochemical experiments were carried out on a Biologic VMP-3e battery cycler (BioLogic, Seyssinet-Pariset, France) in a climatic chamber at 20 ± 1 °C. The three-electrode Swagelok-type cells were used for cyclic voltammetry tests, and two-electrode Swagelok-type cells were used for the rate capability and cycling stability tests. The model cells were mounted in a glovebox (MB-Unilab Pro SP (1500/780) with H_2_O and O_2_ content <0.1 ppm, MBraun, Garching, Germany). Clean sodium metal (Sigma Aldrich, St. Louis, MO, USA) was used as a counter and reference electrode. The electrolyte at 1 M NaPF_6_ in PC (both from Sigma Aldrich, St. Louis, MO, USA) was soaked in glass microfiber separators (Whatman GF/D). To ensure the accuracy of the measurement, each electrochemical experiment was repeated at least twice.

For ex situ XPS experiments, the cells were dissembled in a glovebox, and the recovered electrodes were further analyzed.

## 3. Results

### 3.1. Formation of Carbons from SCGs

The elemental analysis data of pristine and carbonized SCGs are listed in Table 1. Pristine SCGs are rich in carbon, oxygen, and hydrogen elements, which is typical for coffee waste [38]. In addition, a low amount of nitrogen and traces of sulfur were also observed. These elements are usually associated not only with the organic components but also with ash–mineral components [29]. In SCG, the ash-forming elements (determined by EDS analysis) are Si, Mg, K, Al, and Ca (see the discussion below).

**Figure 2:**
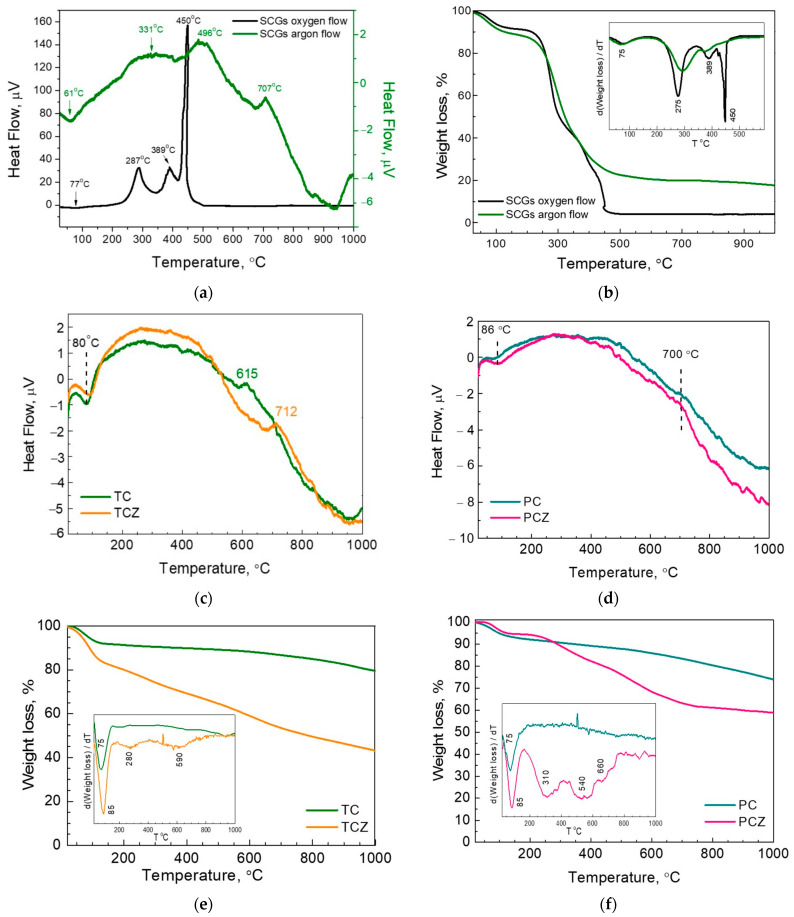
DTA (**a**) and TG (**b**) curves of SCGs in oxygen and argon atmospheres. DTA (**c**,**d**) and TG (**e**,**f**) curves of pre-carbonized samples, TC-8, TCZ-8, PC-8, and PCZ-8 samples in an argon atmosphere. The insets show the first derivative of the corresponding TG curves.

Because of the higher amount of oxygen and hydrogen, SCGs are easily decomposed in both an oxidizing and inert atmosphere (Figure 2). In oxygen flow, the DTA curve shows one endo process at around 77 °C, followed by strong exo processes at around 287, 389, and 450 °C. All thermal processes are accompanied by a corresponding mass loss; for up to 150 °C, the mass loss is 10 m%, and between 200 and 900 °C the mass loss reaches about 86 m.%. (For better resolution, the TG curve is presented in the form of the first derivative, inset Figure 2). Based on previous studies on the thermal behavior of coffee waste [39,40,41], the low-temperature endo process can be assigned to the evaporation of moisture and volatile compounds. The moisture calculated from TG analysis coincides well with that determined from TAPPI standards (Table S1), thus confirming once again the origin of the DTA/TG peak at 75 °C. The next exo processes at 287 and 389 °C can be attributed to the hemicellulose and cellulose degradation process, while the last exothermic process at 450 °C comes from the thermal decomposition of lignin. The higher thermal stability of the lignin than that of hemicellulose and cellulose is a result of its chemical composition: the lignin is richer in aromatic constituents and poorer in oxygen groups [42]. At 900 °C, the solid residue determined from TG analysis is 4 m.%. This value matches the ash content determined by the TAPPI T-211 standard [37] (i.e., ash content of 2.1 m.%, Table S1). According to the XRD analysis (Figure S1), SCG-derived ash consists of a phase mixture between albite calcian low (Na_1_._0–0_._9_Ca_0_._1–0_._3_Al_1_._0–1_._1_Si_3_._0–2_._9_O_8_), MgO, K_2_SO_4_ and Ca_5_(PO_4_)_3_(OH,Cl,F) (Figure S1). A similar phase composition has been reported by Nieto et al. [21] in raw spent coffee grounds as follows: K_2_SO_4_, MgO, Ca_5_(PO_4_)_3_(OH,Cl,F), and CaCO_3_.

When the thermal process takes place in an inert Ar atmosphere, the low-temperature endothermic effect (i.e., peak at ~61 °C and a mass loss of 11 m.%) is preserved, while only two exothermic effects appear at around 290 and 330 °C. The endothermic effect corresponds to moisture evaporation, while exothermic effects reflect the pyrolysis of hemicellulose and cellulose components [43,44]. The exothermic effect at around 707 °C can be explained by a release of some functional groups in cellulose residue [44]. At 900 °C, the residual mass of SCGs is 17.61%, which is higher than that determined in oxygen flow (i.e., of 4.06 wt.%).

Based on the chemical analysis and thermal properties of SCGs, we adopted a synthetic procedure including the pre-carbonization of SCGs at 750 °C, followed by the chemical treatment of pre-carbonized samples and, finally, the annealing of samples at 1100 °C. When pre-carbonization was performed in a fixed bed and in CO_2_ flow, both the oxygen and hydrogen content decreased drastically, while the N-content appeared unchanged (Table 1). It is interesting that the O and H amounts did not show any dependence on the pre-carbonization at 750 °C. The chemical treatment with HCl, HNO_3_, and NaOH yields a complete dissolution of ash containing albite and MgO phases (Table 1) [45,46]. This causes an increase in the oxygen content, thus indicating the procedure of side reactions between pre-carbonized samples and chemical reagents.

The annealing of pre-carbonized TC-8 and PC-8 at 1100 °C leads to a further decrease in the H content, but the O content seems unchanged. The same behavior in H and O content after annealing was observed by Lin et al. [47]: the decrease in hydrogen content was associated with the oxidation of alkyl groups, while the increase in the oxygen content was a result of the formation of C=O groups. In addition to the H and O content, the nitrogen content was also reduced after annealing at 1100 °C. In comparison with pre-carbonized TC-8 and PC-8, the annealing of chemically treated TCZ-8 and PCZ-8 samples produces a drastic decrease in the O content (more than two times), as a result of which this content becomes nearly the same for all types of samples. Moreover, after annealing the H and N content for ash-free PCZ-8 and TCZ-8 samples, these are also comparable to that of ash-containing ones. 

The changes in the chemical composition of pre-carbonized samples with and without ash were further monitored by DTA and TG experiments (Figure 2). Up to 150 °C, all samples lost about 10 w.% due to the evaporation of moisture and volatile matters. On the other hand, this implies that the pre-carbonized samples easily absorb moisture, which is insensitive to their ash content. The availability of mineral ash in SCG-derived samples has an effect on their pyrolysis. Between 150 and 1000 °C, the pre-carbonized TC-8 and PC-8 samples lose an additional 10–15 wt.% without the release of measurable DTA heat. At 1000 °C, the total yield after pyrolysis is around 80–85%. For ash-free pre-carbonized samples, the weight loss between 150 and 1000 °C is more than two times that of the ash-containing samples (Table S1). The close inspection of the TG curve of TCZ-8 signifies that the loss takes place at two stages, 280 and 590 °C, respectively, while for the PCZ-8, at least three stages can be distinguished at 310, 540, and 660 °C. The enhanced mass loss of the ash-free samples correlates with their increased O content after chemical treatment (Table 1). It is of importance that the total yield after pyrolysis reaches around 50 wt.% irrespective of the observed differences in TG curves of TCZ-8 and PCZ-8.

**Figure 3:**
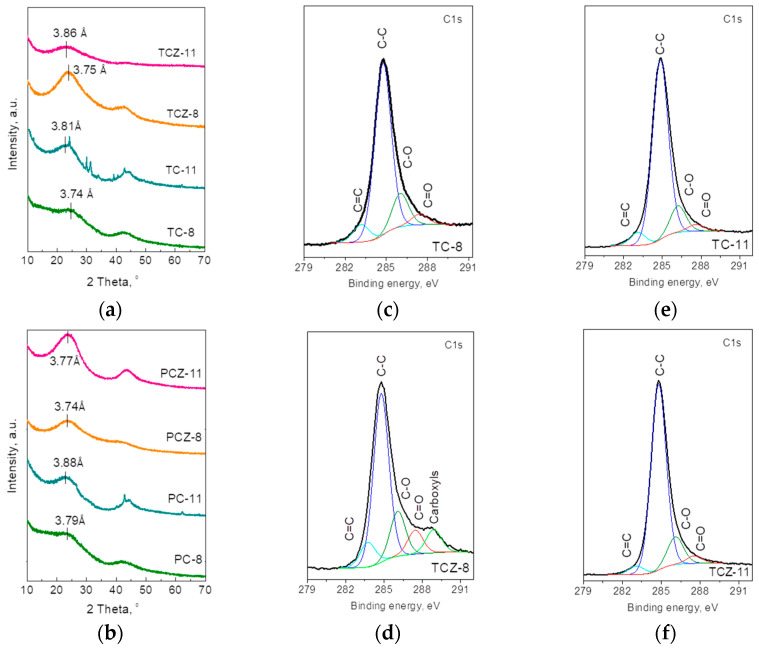
XRD patterns of pyrolyzed SCGs in (**a**) a fixed bed with and without ash, TC-8 and TCZ-8, and their analogs after high-temperature pyrolysis, TC-11 and TCZ-11; (**b**) CO2 flow SCGs with and without ash PC-8 and PCZ-8, and their analogs after high-temperature pyrolysis, PC-11 and PCZ-11. XPS spectra in the energy regions of C 1s for pyrolyzed SCGs obtained in a fixed bed with and without ash (**c**,**d**) and their high-temperature analogs (**e**,**f**).

The structure of SCG-derived samples is assessed by means of X-ray diffraction. Figure 3a,b compares the XRD patterns of the pre-carbonized and pyrolyzed samples. The XRD patterns of all samples display two broad diffraction peaks at approximately 2θ = 23° and 44°, which correspond to the reflections of the (002) and (100)/(101) graphite planes. It is worth mentioning that these XRD features are typical for disordered carbons obtained from biomass [6,13,47]. The (002) reflection expressing the distance between carbon layers has a magnitude of 3.74 Å for all pre-carbonized samples. By increasing the pyrolyzing temperature from 750 to 1000 °C, the *d*-spacing shows a tendency to increase, reaching a value of 3.81–3.88 Å. This means that interlayer space adopts a similar magnitude that is insensitive to the method of pyrolysis. It is of importance that d-spacing is of benefit to the insertion and extraction of Na- ions since it is larger than the required minimum of 3.70 Å [48]. For the ash-containing carbons TC-11 and PC-11, additional narrow peaks were observed, which correspond to the contamination of mineral ash containing albite calcian low, MgO, and K_2_SO_4_. The detection of these crystalline phases at 1100 °C serves as a sign for their formation as secondary products during the pyrolysis of organic components of SCGs; because of the relatively low melting temperature (i.e., 1100–1120 °C), the albite easily crystallizes in the carbonized product. This is a consequence of the high amount of the Si-element in pristine SCGs [29,49]. It is worth mentioning that biomass rich in Si includes coffee waste, rice husks, wood, etc., while soybean husks, grapevine waste, and sunflower pellets and stalks contain a low content of Si [50,51].

The functional groups formed during the carbonization of SCGs are accessed by means of XPS analysis (Figure 3 and Figure S2). The C 1s spectrum of pre-carbonized samples with and without ash consists of an intensive peak at 284.8 eV, which is attributed to the sp^2^-hybridized graphitic carbon [52,53]. Superimposed to the peak at 284.8 eV, three less intensive peaks at (286.1 ± 0.2)  eV, (287.4 ± 0.2) eV and (288.8 ± 0.2) eV can be distinguished. Although the peak at 286.1 eV can be assigned to C atoms bonded to oxygen in hydroxyl/epoxide groups (C–O), the carbonyl and carboxyl groups (C=O and O–C=O) give rise to the peaks at 287.4 eV and 288.8 [46,52]. The carboxyl groups appear only for the ash-free samples TCZ-8 and PCZ-8. Supporting this assignment of the C 1s signals, the O 1s spectra of pre-carbonized samples can be deconvoluted for at least three peaks at 529.0, 531.5, and 533.0 eV. The peak at 529.0 eV is adequately resolved for the ash-containing TC-8 and PC-8 and can be assigned to the O atoms bonded to ash elements such as Si, Mg, and K. The peak at 531.5 eV comes, most probably, from the double-bonded O atoms in esters and/or acids, while the single-bonded O atoms in ketons and/or ethers are responsible for the peak at 533.0 eV [54,55]. The functional C–O and C=O groups remain stable even after pyrolysis at 1100 °C. Moreover, the functional groups seem insensitive towards the method of carbonization, as well as towards ash removal.

**Figure 4:**
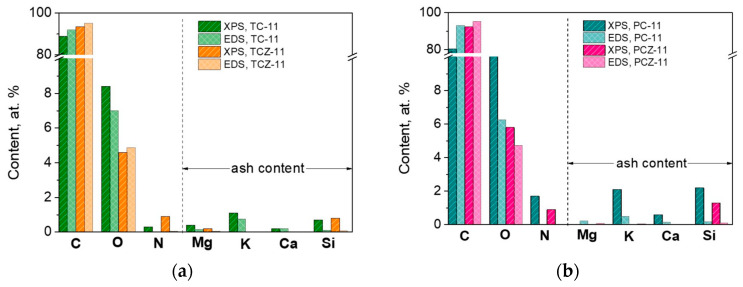
The concentration of elements determined by XPS and EDS analysis obtained after high-temperature pyrolysis and being pre-carbonized in a fixed bed (**a**) and CO2 flow (**b**).

The only parameter that is changed after carbonization is the elements’ concentration. Figure 4 compares the element concentration determined by XPS and EDS analysis. These techniques are used in a complementary way to gain access to the distribution of elements along the depth of the carbonized samples at 1100 °C; the XPS method permits the identification and quantification of elements located on the outermost surface layers (i.e., up to 5 nm), while the EDS provides information on elements located inside the thicker layers (more than 500 nm). The data disclose that the oxygen is inhomogeneously distributed along the depth of particles, especially for ash-containing TC-11 and PC-11: the oxygen content is higher on the surface than the inside of the volume. For the ash-free samples, the oxygen is more or less homogeneously distributed. Furthermore, the amount of ash elements determined by XPS is higher than those evaluated by EDS. (The XPS spectra of Si 2p are given in Figure S3). This is a mark that mineral ash accumulates mainly on the surface of carbonized products, this being insensitive towards the method of pre-carbonization. In analogy with ash elements, nitrogen is also exclusively located on the surface.

The morphology of SCG-derived carbons was monitored by scanning electron microscopy, and the corresponding SEM images are shown in Figure S4. For ash-containing TC-11, the morphology consists of plate-like particles packed in columns, the aggregate sizes being around 1.5–2.5 µm. After ash removal, plate-like aggregates retain, in general, their sizes, but the degree of aggregate packing seems to diminish. In comparison to TC-11, the PC-11 displays plate-like aggregates with a more inhomogeneous size distribution spreading from 0.5 to 3.0 µm. The removal of ash from PC-11 does not have a significant effect on the carbon morphology.

**Figure 5:**
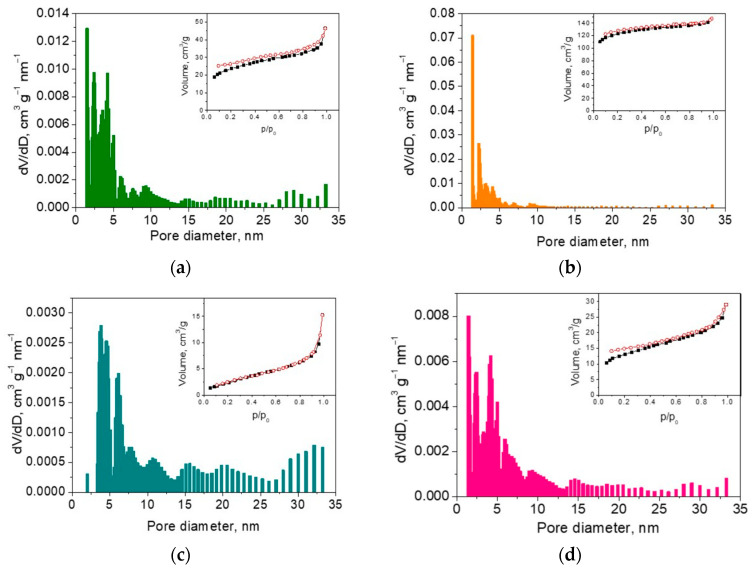
NLDFT pore size distribution of (**a**) TC-11, (**b**) TCZ-11, (**c**) PC-11, and (**d**) PCZ-11. The corresponding nitrogen adsorption–desorption isotherms are shown as insets.

The texture properties of SCG-derived carbons enable us to differentiate with respect to the method of carbonization and ash removal (Figure 5 and Figure S5). The sample TC-8 obtained in a fixed bed has a low specific surface area of 17 m^2^ g^−1^, while after ash removal, the specific surface area increases more than 10 times (i.e., up to 409 m^2^ g^−1^) (Table 2). Irrespective of the observed change in the specific surface area, both TC-8 and TCZ-8 possess isotherms classified as I-type (Figure S5a,b, insets), which is characteristic of microporous materials. The pore size distribution and NLDFT calculation show that besides micropores, both carbons have mesopores with sizes between 5 and 7 nm. After the high-temperature pyrolysis, the specific surface area additionally increases, especially for the ash-free sample TCZ-11 (Table 2). Even in this case, TCZ-11 still exhibits an isotherm of the I-type, and the ratio between micro- and mesopore volumes Vmi/Vmes remains nearly unchanged (Figure 5b, inset). In comparison with the ash-free sample, the ash-containing sample shows a different behavior after high-temperature pyrolysis: the isotherm is changed from the I- to the H4-type [56,57], which is associated with the occurrence of narrow slit pores. The H4-type isotherm is often observed for carbons obtained from lignin-rich biomass [6,47]. The specific surface area of TC-11 also increases, but the ratio Vmi/Vmes decreases (Table 2), thus indicating the formation of more mesopores (Figure 5a).

In comparison with TC-8 and TCZ-8, the samples obtained in CO_2_ flow (i.e., PC-8 and PCZ-8) exhibit isotherms belonging to the H3-type (Figure S5c, inset). These isotherms with a specific hysteresis loop are typical for materials with non-rigid aggregates of plate-like particles characterized by unlimited adsorption at high p/po [56,57]. After high-temperature pyrolysis at 1100 °C, the isotherm of PC-11 is transformed from the H3 to the II-type (Figure 5c), thus revealing a formation of a nonporous or macroporous-carbonized sample. The NLDFT calculation reveals a broad pore size distribution—from ~2.5 to ~33 nm (Figure 5c)—with a large quantity of mesopores (Table 2). Contrary to PC-11, the transformation of the isotherm for PCZ-11 is from H3 to H4 (Figure S5d, inset). The hysteresis loop is associated with narrow slip-shaped pores.

In general, the comparison between the ash-containing and ash-free analogs (i.e., TC-11 and TCZ-11, as well as PC-11 and PCZ-11) shows that the specific surface area increases after the ash removal, while the amount of mesopores is significantly greater for ash-containing samples (Table 2, Figure 5 and Figure S5).

### 3.2. Sodium Storage Performance of SCG-Derived Carbons

The capability of SCG-derived carbons to store Na is evaluated by CV measurements at a potential range of 0.05–2.0 V and a scanning rate varying from 0.01 mV s^−1^ to 100 mV s^−1^ (Figure S6). For all the samples, the first cathodic scan at a rate of 0.01 mV s^−1^ displays a broad wave between 0.9 and 0.3 V, which is not restored during the reverse anodic scan. This wave is assigned to the decomposition of the electrolyte, leading to the formation of the solid electrolyte interphase (SEI) on the electrode surface [35,36,48]. The calculated capacitance corresponding to the first cathodic scan between 2.0 and 0.3 V is shown in Figure S6b. This comparison provides evidence that PC samples exhibit higher irreversible capacitance than TC analogs. This means that a thicker SEI is formed on the surface of PC samples. The thicker SEI for the PC samples could be related to their broader pore size distribution [58] (Figure S6). In addition, the effect of the ash content is small. On the other hand, this can serve as an indirect sign that the specific surface area is not a leading factor for the conditioning of SEI: the ash-free samples have the highest specific surface area (Table 2), but the biggest capacitance is determined for the ash-containing sample PC-11. This is in agreement with previous studies, where the contributions of a specific surface area and pore size distribution in the SEI formation have been demonstrated [59].

**Figure 6:**
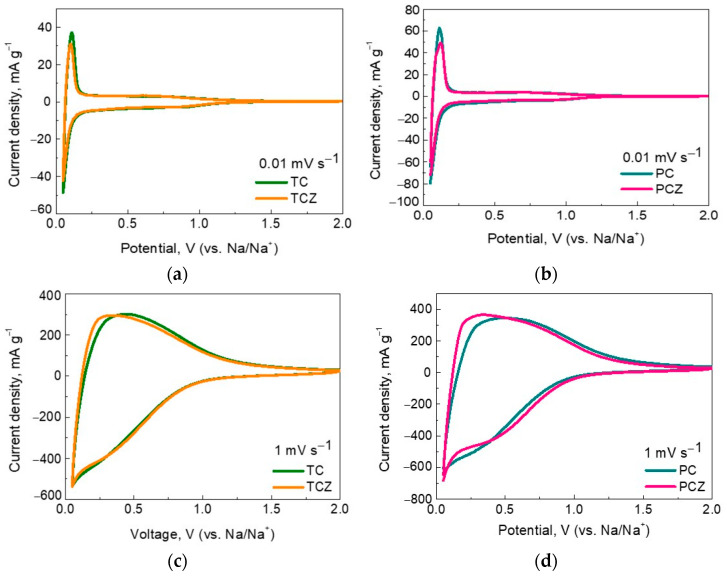
CV curves of SCG-derived carbon at 0.01 mV s^−1^ (**a**,**b**) and 1 mV s^−1^ (**c**,**d**).

After the first irreversible cathodic scan, the CV curves adopt stable profiles, where sharp redox peaks below 0.1 V are clearly observed at slow scanning rates (Figure 6 and Figure S7). These peaks can be associated with the reversible intercalation of Na^+^ between graphitic layers and their further adsorption in the micropores [60]. By increasing the scan rate from 0.01 mV s^−1^ to 1 mV s^−1^, the very broad redox wave centered at around 0.25 V becomes dominant at the expense of the sharp redox peaks. This wave comes from the adsorption-induced Na^+^ storage on the pores and defect sites of hard carbons [48]. At the highest scan rate (i.e., 100 mV s^−1^), the CV profiles resemble those of capacitive reactions (Figure S7). The established dependence of the Na^+^ intercalation and adsorption reactions on the scan rate corroborates with previous data on the Na^+^ adsorption energy and diffusion barrier: Na^+^ adsorption proceeds faster than Na^+^ intercalation [61]. The important finding is that all of the SCG-derived carbons have the same CV features, thus revealing a constancy in the Na storage mechanism irrespective of the carbonization procedure, as well as the ash content.

The galvanostatic experiments further support the main features of the Na storage mechanism. The SEI on carbons is formed after the first cycle, the measure of which is the first irreversible capacity. The highest magnitude of the first irreversible capacity reaches the PC samples (208 and 147 mAh g^−1^ corresponding to ICE 49% and 60% for PC-11 and PCZ-11, respectively), thus confirming the growth of a thicker SEI on them in comparison with the TC samples (119 and 145 mAh g^−1^ corresponding to ICE 56% and 52% for TC-11 and TCZ-11, respectively). This trend is valid irrespective of the ash content. It is noticeable that the first irreversible capacity follows the order already established from CV data (Figure 6 and Figure S7). The comparison of the galvanostatic and CV data evidence that the ash content slightly affects the SEI formation, while the manner of pore size distribution is a main factor contributing to SEI’s growth.

**Figure 7:**
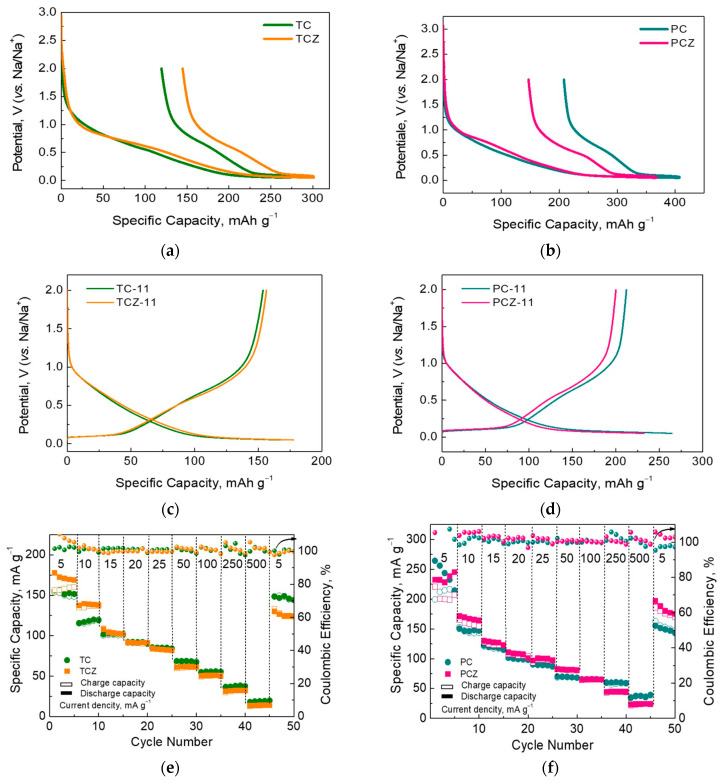
Initial discharge and reversible charge capacity of high-temperature samples with and without ash obtained in a fixed bed, TC and TCZ (**a**) and in CO_2_-flow, PC and PCZ (**b**); original charge–discharge curves for the second cycle of TC/Z (**c**) and PC/Z (**d**); rate capability TC/Z (**e**) and PC/Z (**f**).

The second cycle enables an evaluation of the reversible capacity delivered by SCG-derived carbons (Figure 7c,d). To avoid sodium plating and reactivity at low potentials in the sodium-half cell [62,63], the low voltage limit is set to 0.05 V. These data show that TC samples with and without ash possess around a 110 mAh g^−1^ capacity due to the Na^+^ adsorption, while the Na^+^ intercalation gives rise to a capacity of around 70 mAh g^−1^, respectively. In general, PC samples deliver high capacity in comparison with TC samples.

The cycling stability and rate capability are the next parameters that enable the differentiation of samples with respect to the ash content (Figure 7e,f). At a lower current load (5 mA g^−1^), ash-free TCZ-11 delivers a slightly higher capacity than that of the ash-containing analog. However, Coulombic efficiency is significantly better for ash-containing TC-11, especially after the first five cycles. By increasing the current load from 5 to 500 mA g^−1^, the capacity of TC-11 gradually becomes higher than that of TCZ-11, thus disclosing the better rate capability of the ash-containing sample. For a whole rate-capability test, the Coulombic efficiency for TC-11 remains more stable than that of TCZ-11. In comparison with TC, the PC samples display the same features: ash-containing PC-11 has a better rate capability and Coulombic efficiency than the ash-free analog PCZ-11. All these data give evidence for the better electrochemical performance of the ash-containing samples than their ash-free analogs. The improved performance could be correlated with the surface modification of the carbonized materials through an accumulation of the mineral ash (namely low albite calcian, MgO, and K_2_SO_4_). This mechanism is different from that established by Zhang et al., where the ash-containing samples display worse cycling stability due to the occupancy of active Na storage sites by mineral impurities [25]. However, the occurrence of impurity potassium in hard carbon obtained from coconut endocarp leads to facilitating Na^+^ diffusion by expanding the interlayer spacing of the graphitic layers [36]. This suggests that the impact of the mineral ash on carbon performance depends not only on the phase composition but also on the manner of ash distribution.

**Figure 8:**
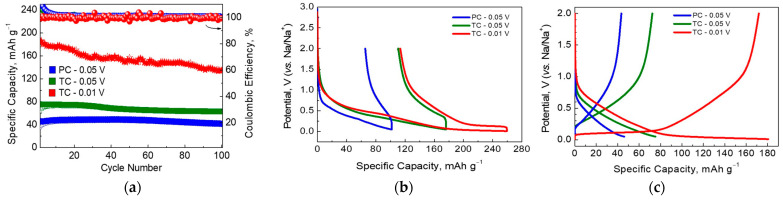
(**a**) Cycling stability at a current load of 50 mA g^−1^ for ash-containing high-temperature samples. (**b**) Charge/discharge curves for ash-containing SCG-derived carbons during the first and (**c**) fifth cycles.

Figure 8 compares the cycling stability of ash-containing TC-11 and PC-11 under a current load of 50 mA g^−1^. Despite the electrochemical inactivity of mineral ash, the comparison shows that TC-11 outperforms PC-11. This implies that the Na storage performance depends both on the carbon structure (which is a result of the carbonization procedure) and ash phases.

Taking into account that most studies provide information on the specific capacity of deeply discharged carbons (i.e., the low voltage limit of 0.01 V or 0.002 V), we performed additional electrochemical tests on TC-11 by decreasing the low voltage limit from 0.05 V to 0.01 V and keeping the upper voltage limit to 2.0 V. Figure 8b shows the charge/discharge curves for “softly” and “deeply” discharged TC-11 after the 1st and 5th cycles under 50 mA g^−1^. By decreasing the low voltage limit, both the discharge and charge capacities increased dramatically, but the irreversible capacity remained the same (i.e., 113 mAh g^−1^). This resulted in an increase in the initial Coulombic efficiency (ICE): from 37 to 56%. After further cycling, the Coulombic efficiency (CE) tended to be 99–100% without any loss of capacity. For the 20th cycle, the specific capacities for “softly” and “deeply” discharged TC-11 were 75 and 168 mAh g^−1^, respectively. To demonstrate the electrochemical performance of ash-containing carbons, Table 3 compares our data with previously reported ones for coffee-derived electrode materials using different preparation methods. The comparison reveals that ash-containing TC-11 displays electrochemical parameters that are quite comparable to carbons undergoing additional chemical treatment. This means that mineral ash has a beneficial effect on the Na storage performance of SCG-derived carbons.

### 3.3. Ex Situ XPS Analysis

**Figure 9:**
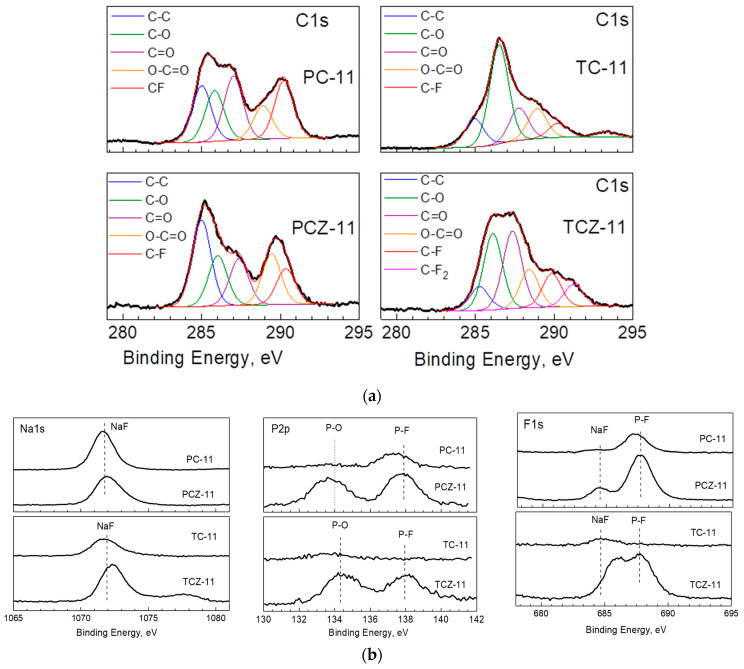
XPS spectra in the energy regions of C 1s of the cycled electrodes for pyrolyzed samples with and without ash obtained in a fixed bed and CO_2_ flow (**a**), as well as Na 1s, P 2p, and F 1s (**b**).

**Figure 10:**
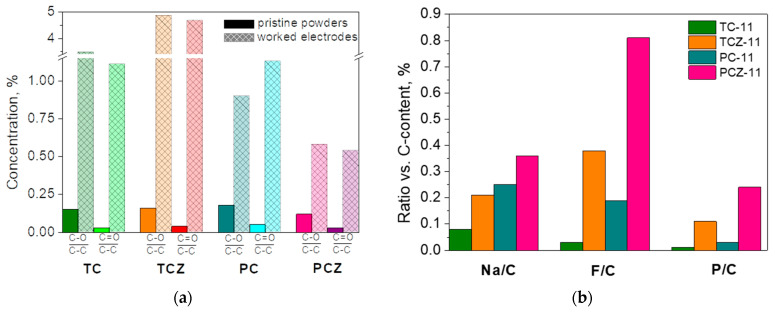
Calculated ratio (C–O)-to-(C–C) and (C=O)-to-(C–C) for the electrodes and pristine samples (**a**). The amount of Na, F, and P elements relative to carbon content (**b**).

To rationalize the performance of carbon-based electrodes, ex situ XPS was undertaken with the aim of assessing the decomposition products forming the SEI. In this aspect, XPS spectra in the binding-energy region of C, Na, F, and P elements were of primary interest (Figure 9). After the electrochemical reaction, the C 1s spectra of all electrodes were dominated by the signals due to C–O, C=O, and O–C=O groups. In addition, the characteristic signals due to the C-F and C-F_2_ groups also appeared, especially for TCZ-11. The detection of these surface products can be regarded as a result of the interaction of the electrode surface with the electrolyte, leading to the deposition of C–O, C=O, and O–C=O and C-F groups. For the sake of better comparison, the calculated ratios (C–O)-to-(C–C) and (C=O)-to-(C–C) for the electrodes and pristine samples are given in Figure 10a. The comparison shows that the C–O and C=O groups increase dramatically after the electrochemical reaction, especially for the TC samples. This means that the electrolyte–electrode interaction leads to the deposition of C–O and C=O groups at higher amounts for the TC-11 and TCZ-11 electrodes in comparison with PC-electrodes.

While the C 1 spectra provide information on the products coming from the electrolyte solvent decomposition (i.e., PC molecules), electrolyte salt, NaPF_6_, and decomposition on the electrode surface is evaluated by Na 1s, F 1s and P 2p spectra (Figure 9b). In the energy region of Na 1s, one broad signal centered at 1072 eV is observed for all carbon electrodes. The binding energies of Na 1s correspond to Na atoms in NaF [64]. The Na 1s signal assignment is supported by the F 1s spectra, where a signal centered at 684.5 eV is clearly resolved, and it is associated with the F atom in NaF [65]. In addition, the F 1 spectra display a second signal at 687.6 eV, which is attributed to F atoms in Na_x_PF_y_O_z_ [65]. In agreement with F 1, the P 2p spectra consist of two overlapping signals centered at 133.7 and 137.7 eV. The signal with a lower binding energy could be assigned to P_2_O_5_ and/or Na_x_PF_y_O_z_, while the signal with a higher binding energy comes from Na_x_PF_y_. It is worth mentioning that the ash-free samples PCZ-11 and TCZ-11 are covered predominately with Na_x_PF_y_O_z_ and Na_x_PF_y_ products, while NaF is the main surface product for the ash-containing samples.

The next characteristics for electrodes determined by ex situ XPS analysis are the amounts of Na, F, and P elements (Figure 10b). The comparison discloses that the surface of PC samples is richer on Na than that of the TC samples. The removal of ash from both TC and PC samples facilitates Na deposition. While the changes in the Na content between TC and PC samples are relatively moderate (between 1.5 and 2 times), the changes in the F and P content are dramatic (more than 3–5 times). As in the case of Na, the surface of PC samples concentrates more F and P elements than that of the TC samples. Furthermore, the F and P elements are predominantly deposed when ash is removed from the carbons. A close inspection of Figure 10 shows that the ash phases significantly reduce the decomposition of NaPF_6_ salt.

Based on ex situ XPS analysis, one can conclude that the SEI of carbon electrodes is composed of organic and inorganic products coming mainly from the decomposition of the electrolyte solvent (i.e., propylene carbonate) and electrolyte salt (i.e., NaPF_6_), with the ratio between them being dependent on the method of carbonization and the ash content. The surface of carbons obtained in a fixed bed is richer on organic decomposition products, while inorganic decomposition products are deposed on the surface of carbons obtained in CO_2_ flow. In comparison with organic products, it appears that inorganic products contribute to a higher extent to the formation of SEI, as a result of which PC samples form a thicker SEI (Figure 10b). Ash-free carbons form thicker SEI than that ash-containing analogs. This implies that ash phases, mainly albite, stimulate the decomposition of the solvent electrolyte molecules and their deposition on the electrode surface. Along with this, ash phases prevent electrolyte salt decomposition.

## 4. Conclusions

This study demonstrates that complementary to the carbon structure and texture, mineral ash phases and their distribution have an impact on the Na storage properties of SCG-derived carbons. Because of the higher amount of Si and Al in SCGs, the carbonized products contain mineral ash composed mainly of low albite calcian, MgO, and K_2_SO_4_. The mineral ash is mainly located on the outmost surface of carbonized products.

The Na storage proceeds the same mechanism for ash-containing and ash-free carbons; for up to 0.1 V, there is Na^+^ adsorption, while below 0.1 V, both Na^+^ intercalation and/or Na^+^ nanopore filling occurs. However, ash-containing carbons outperform ash-free analogs with respect to cycling stability, Coulombic efficiency, and rate capability. At a current load of 50 mA g^−1^, the “softly” and “deeply” discharged carbons (i.e., up to 0.05 and 0.01 V) deliver a reversible capacity of 75 and 168 mAh g^−1^. The better performance of ash-containing carbons can be explained by the electrode–electrolyte interaction. The SEI of ash-containing carbons is mainly composed of organic products resulting from electrolyte solvent decomposition. The electrolyte salt decomposition is suppressed by the presence of the ash phases. The SEI richer in inorganic decomposition products is preferentially formed on carbons obtained in a CO_2_ flow.

In general, the presence of mineral ash and its distribution on the carbon surface helps to improve the performance of SCG-derived carbons. This study reveals that there is no need to utilize additional and costly procedures for the removal of mineral ash.

## Figures and Tables

**Table 1:** Elemental analysis of pristine SCGs and carbons after low-temperature annealing (750 °C) and high-temperature pyrolysis (1100 °C).

Sample	C, Mass %	H, Mass %	N, Mass %	S, Mass %	O, Difference	O/C
SCGs	49.91	7.41	2.83	0.16	39.66	0.79
TC-8	77.74	2.19	4.27	0.24	15.56	0.20
TC-11	79.75	0.85	1.02	0.18	19.05	0.24
TCZ-8	57.13	2.25	3.68	0.19	36.75	0.64
TCZ-11	80.93	1.41	1.75	0.17	15.74	0.19
PC-8	74.82	2.19	3.70	0.21	19.08	0.26
PC-11	80.58	0.94	0.46	0.15	17.87	0.22
PCZ-8	51.09	2.54	3.94	0.19	42.24	0.83
PCZ-11	78.42	1.43	1.70	0.15	18.30	0.23

**Table 2:** BET specific surface area, total pore volume, average pore size, micropore volume, and volume ratio between the micro- and mesopores.

Samples	S, m^2^ g^−1^	V, cm^3^ g^−1^	Dav, nm	Vmi, cm^3^ g^−1^	Vmi/Vmes
TC-8	17	0.008	2.00	0.006	3
TC-11	86	0.07	1.9	0.02	0.4
TCZ-8	409	0.20	1.9	0.14	2.33
TCZ-11	471	0.23	1.7	0.16	2.3
PC-8	10	0.001	3.2	-	-
PC-11	10	0.02	2.9	0.006	0.31
PCZ-8	8	0.008	3.2	0.001	0.14
PCZ-11	48	0.05	1.9	0.03	1.5

**Table 3:** Comparison of the electrochemical parameters of coffee-derived carbons.

Bio-Waste	Synthesis Procedure	Temperature Obtained	Current Density	Potential Range, (vs. Na/Na^+^)	ICE, %	First Cycle Reversible Capacity, mAh/g	Ref.
Spent coffee grounds	(1) Hydrothermal synthesis (HtS); (2) Chemical treatment after HtS; (3) One-step pyrolysis in Ar-flow.	700 °C	50 mA/g	0.01–3.00 V	55	198	[15]
Coffee grounds (CGs)	(1) Pre-treatment of CGs with KOH for 24 h; (2) One-step carbonization in N_2_.	900 °C	50 mA/g	0.01–3.00 V	64	190 (untreated)	[19]
					39	144 (treated with KOH 1:1)	
Coffee grounds (CGs)	(1) Pre-treatment of CGs with HCl for 3 days; (2) One-step pyrolysis in Ar-flow.	970 °C	C/5 (1C = 300 mAh/g)	0.02–2.2 V	~39	~130 (CMC binder)	[20]
Spent Coffee Grounds	One-step carbonization in N_2_.	1200 °C	C/15 (1C = 372 mAh/g)	0.002−2.0 V	34	199 (untreated)	[21]
	(1) Hydrothermal pretreatment (HTC); (2) One-step pyrolysis in N_2_.				32	219 (HTC pre-treated)	
Waste beverage coffee (WBC)	Two-step pyrolysis (first step at 800 °C in N_2_ and the second in Ar).	1600 °C	10 mA/g	0.01−2.0 V	56	~168	[22]
		2000 °C			90	~270	
		2400 °C			75	~225	
		2600 °C			64	~194	
Our samples—spent coffee grounds	Two step pyrolysis in a fixed bed (TC) at 750 °C and 1100 °C in Ar flow.	1100 °C	50 mA/g	0.05−2.0 V	37	65	
				0.01−2.0 V	56	146	

## Data Availability

Data are contained within the article and Supplementary Materials.

## Supplementary Materials

The following supporting information can be downloaded at: https://www.mdpi.com/article/10.3390/ma17051016/s1, Table S1: Moisture and ash content in the SCGs and carbon yields after pyrolysis; Figure S1: XRD pattern of the SCG-derived ash at 525 °C; Figure S2: XPS spectra in the energy regions of C1s for pyrolyzed SCGs obtained in CO_2_ flow with and without ash (a,b) and their high-temperature analogs (c,d); Figure S3: XPS spectra in the energy regions of O 1s and Si 2p for pyrolyzed SCGs in a fixed bed (a,b) and in CO_2_ flow (c,d), respectively; Figure S4: SEM images of (a) TC-11, (b) TCZ-11, (c) PC-11, and (d) PCZ-11 obtained after high-temperature pyrolysis; Figure S5: NLDFT pore size distribution of the (a) TC-8, (b) TCZ-8, (c) PC-8, (d) PCZ-8. The corresponding nitrogen adsorption–desorption isotherms are shown as insets; Figure S6: CV curves during the first cathodic cycle at 0.01 mV s^−1^ scan rate (a) and the calculated capacitance in the potential window 2.0–0.3 V (b); Figure S7: CV curves of SCG-derived carbon at 100 mV s^−1^.
