# Supplementary material for: The Beneficial Impact of Mineral Content in Spent-Coffee-Ground-Derived Hard Carbon on Sodium-Ion Storage
Source: Materials (Basel). 2024 Feb 22;17(5):1016. doi: 10.3390/ma17051016 (PMC10935302; doi:10.3390/ma17051016)
Supplement: Supplementary file 1 [file materials-17-01016-s001.zip › materials-2824979-supplementary.pdf]

Supplementary Materials

The Beneficial Impact of Mineral Content in Spent-Coffee-Ground-Derived Hard Carbon on Sodium-Ion Storage

Sonya Harizanova, Ivan Uzunov, Lyubomir Aleksandrov, Maria Shipochka, Ivanka Spassova and Mariya Kalapsazova *

Institute of General and Inorganic Chemistry, Bulgarian Academy of Sciences, 4000 Plovdiv, Bulgaria; sonya@svr.igic.bas.bg (S.H.); uzunov_iv@svr.igic.bas.bg (I.U.); lubomir@svr.igic.bas.bg (L.A.); shipochka@svr.igic.bas.bg (M.S.); ispasova@svr.igic.bas.bg (I.S.)

* Correspondence: maria_l_k@svr.igic.bas.bg

Table S1. Moisture and ash content in the SCGs, and carbon yields after pyrolysis.

	Coffee		TC	TCZ	PC	PCZ
	O_2	Ar	Ar			
Miosture TAPPI T550, m. %	8.1					
Miosture DTA/TGA, m. %	10	11				
Volatile matters DTA/TGA, m %	86	71				
Solid residue DTA/TGA, m. %	4	18				
Ash content TAPPI T211, m. %	2.1					
Mass loss DTA/TGA, m. %			21	58	27	42
Mass loss after pyrolysis, m. %			18	48	17	55
Yield after pyrolysis, m. %			82	51	82	45

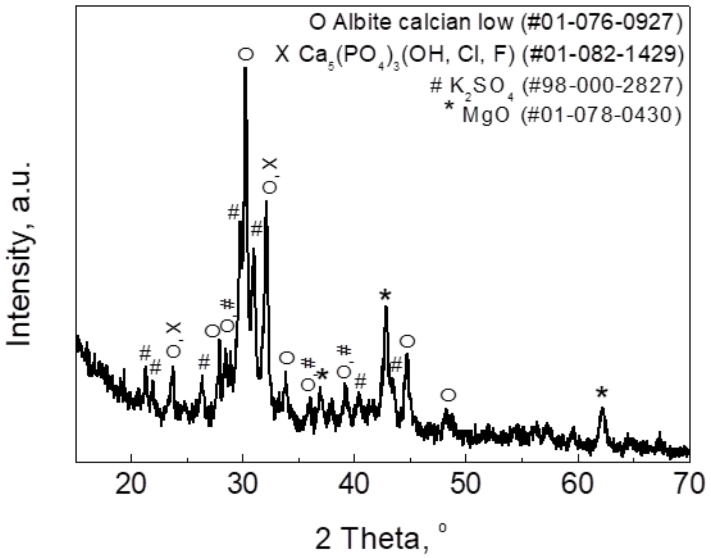

Figure S1. XRD pattern of the SCG-derived ash at 525 °C.

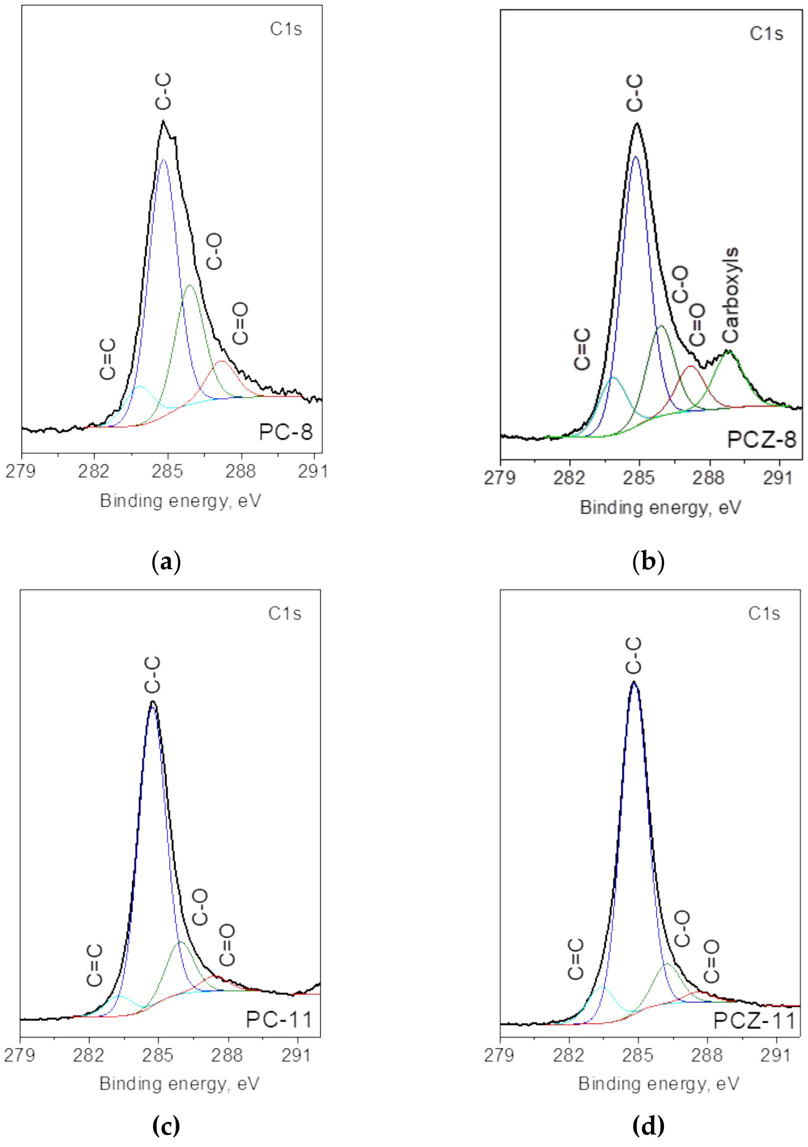

Figure S2. XPS spectra in the energy regions of C1s for pyrolyzed SCGs obtained in CO_2 flow with and without ash (a, b) and their high-temperature analogues (c, d).

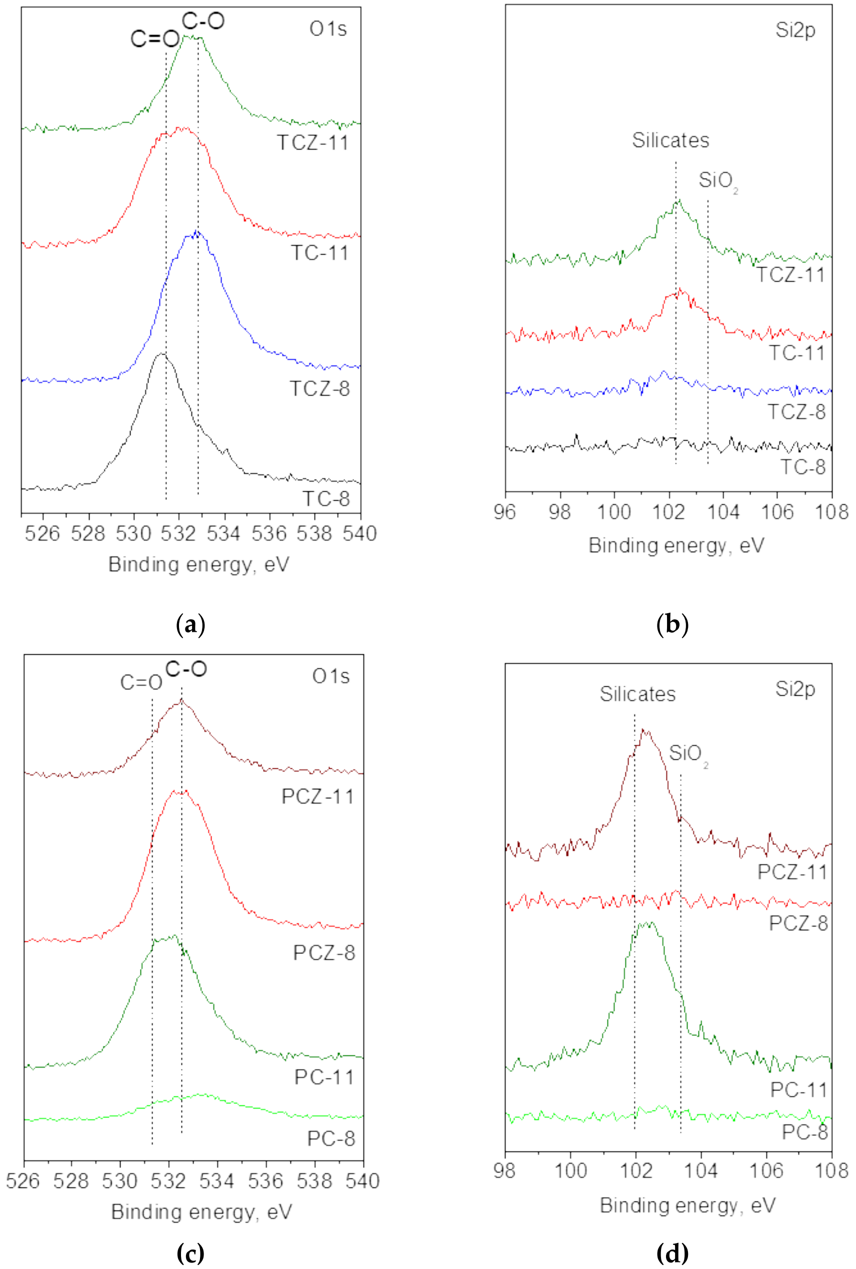

Figure S3. XPS spectra in the energy regions of O 1s and Si 2p for pyrolyzed SCGs in fixed-bed (a, b) and in CO_2 flow (c, d), respectively.

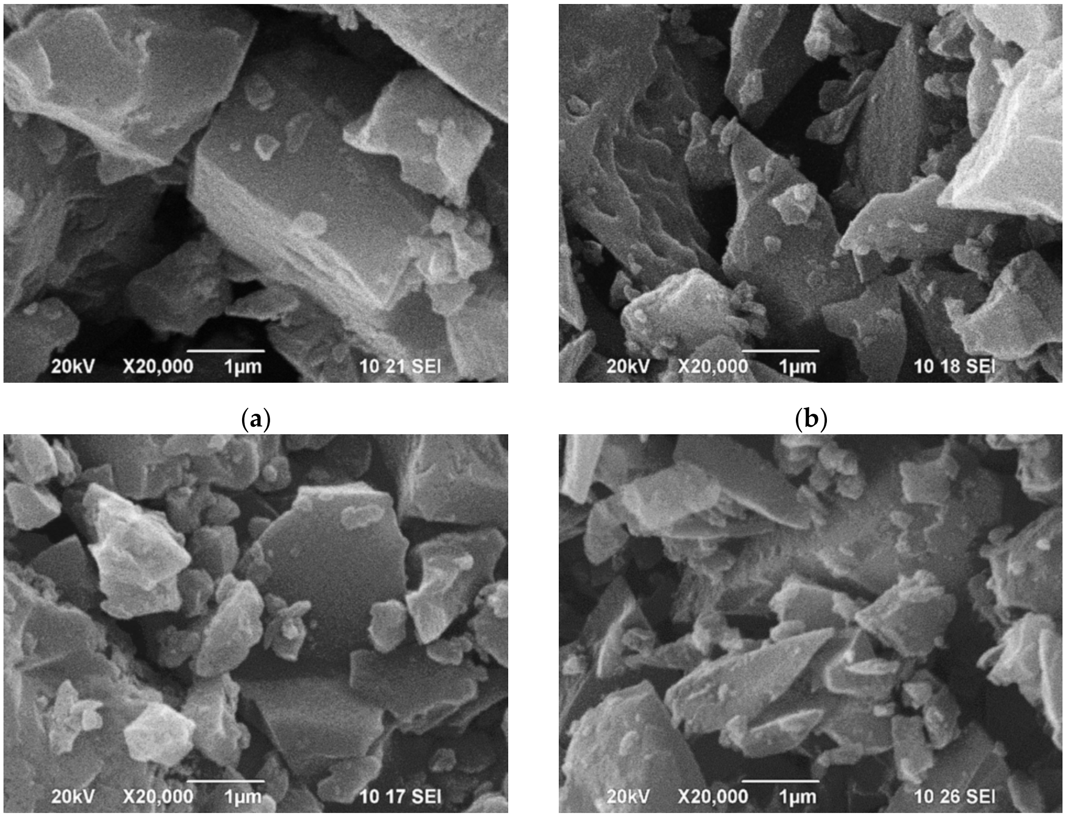

(**a**) (**b**)

(c) **(d)**

Figure S4. SEM images of (a) TC-11, (b) TCZ-11, (c) PC-11, and (d) PCZ-11 obtained after high-temperature pyrolysis.

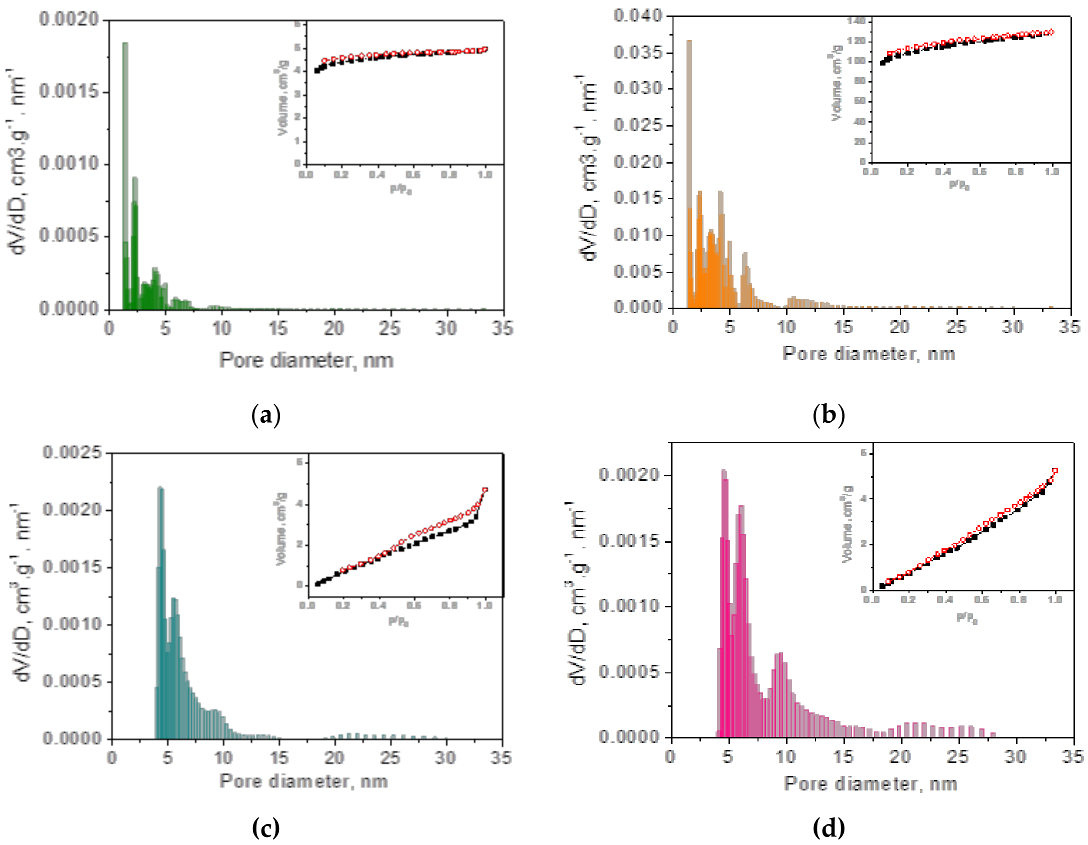

Figure S5. NLDFT pore size distribution of the (a) TC-8, (b) TCZ-8, (c) PC-8, (d) PCZ-8. The corresponding nitrogen adsorption-desorption isotherms are shown as insets.

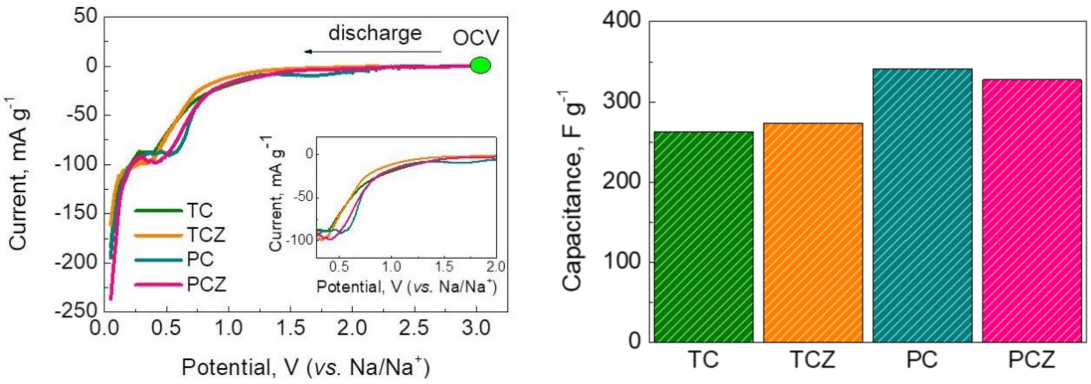

(**a**) (**b**)

Figure S6. CV curves during the first cathodic cycle at 0.01 mV s^{-1} scan rate (a) and the calculated capacitance in the potential window 2.0 - 0.3 V (b).

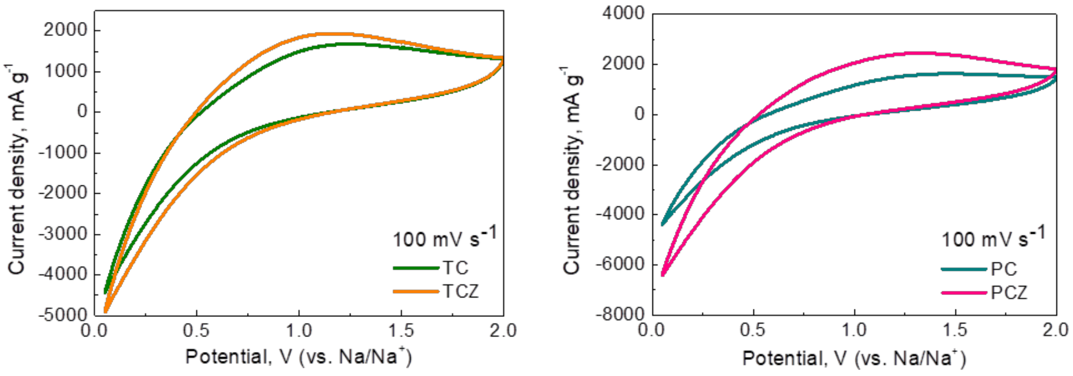

(**a**) (**b**)

Figure S7. CV curves of the SCG derived carbon at 100 mV s^{-1}.
